# The TIPE Molecular Pilot That Directs Lymphocyte Migration in Health and Inflammation

**DOI:** 10.1038/s41598-020-63629-w

**Published:** 2020-04-20

**Authors:** Honghong Sun, Mei Lin, Ali Zamani, Jason R. Goldsmith, Amanda E. Boggs, Mingyue Li, Chin-Nien Lee, Xu Chen, Xinyuan Li, Ting Li, Brigid L. Dorrity, Ning Li, Yunwei Lou, Songlin Shi, Wei Wang, Youhai H. Chen

**Affiliations:** Department of Pathology and Laboratory Medicine, Perelman School of Medicine, University of Pennsylvania, Philadelphia, PA USA

**Keywords:** Cell biology, Immunology, Pathogenesis

## Abstract

Lymphocytes are some of the most motile cells of vertebrates, constantly navigating through various organ systems. Their specific positioning in the body is delicately controlled by site-specific directional cues such as chemokines. While it has long been suspected that an intrinsic molecular pilot, akin to a ship’s pilot, guides lymphocyte navigation, the nature of this pilot is unknown. Here we show that the TIPE (TNF-α-induced protein 8-like) family of proteins pilot lymphocytes by steering them toward chemokines. TIPE proteins are carriers of lipid second messengers. They mediate chemokine-induced local generation of phosphoinositide second messengers, but inhibit global activation of the small GTPase Rac. TIPE-deficient T lymphocytes are completely pilot-less: they are unable to migrate toward chemokines despite their normal ability to move randomly. As a consequence, TIPE-deficient mice have a marked defect in positioning their T lymphocytes to various tissues, both at the steady-state and during inflammation. Thus, TIPE proteins pilot lymphocytes during migration and may be targeted for the treatment of lymphocyte-related disorders.

## Introduction

Leukocyte migration in response to chemical attractants is essential for immune homeostasis, immunity, and inflammation^1–4^. Drugs blocking leukocyte migration (such as the FDA-approved natalizumab and fingolimod) are highly effective for treating human inflammatory diseases such as multiple sclerosis^5,6^. However, how leukocytes sense and follow shallow chemical gradients during homing or inflammation is not well understood. In order for cells to move in one direction, they must first form a defined front (leading edge) and rear (trailing edge). This front-rear polarity is characterized by asymmetrical activation of proteins such as phosphoinositide 3-kinases (PI3Ks), GTPase Rac, and actin regulatory proteins at the leading and trailing edges. How shallow gradients reliably translate into axes of cell polarity is poorly understood^7,8^. Several computational models have been proposed to address this issue. The LEGI-BEN (local-excitation, global-inhibition - biased excitable network) model predicts that both enhancers and inhibitors of signal transduction are required for leukocyte polarization and chemotaxis^9,10^. The enhancers operate locally at the leading edge, whereas the inhibitors function globally. However, the nature of these enhancers and inhibitors is not well understood.

The TIPE (tumor necrosis factor-α-induced protein 8 (TNFAIP8)-like, or TNFAIP8L) family of proteins are newly discovered regulators of inflammation and cancer. There are four highly homologous mammalian TIPE family members: TNFAIP8, TIPE1 (TNFAIP8L1), TIPE2 (TNFAIP8L2), and TIPE3 (TNFAIP8L3). TNFAIP8 overexpression is associated with increased tumor metastasis^11–13^, and *TNFAIP8* gene single nucleotide polymorphisms (SNPs) are associated with the development of several inflammatory diseases including inflammatory bowel disease (IBD) with pyoderma gangrenosum^14^, multiple sclerosis^15^ and plantar fasciitis^16^, as revealed from recent genome-wide association studies (GWAS). Similarly, TIPE2 regulates both innate and adaptive immunity, and is a risk factor for IBD, and one of the key driver genes that can cause IBD as revealed from the “functional genomics predictive network model” of IBD^17^. Of the four members of the mammalian TIPE family, TIPE2 and TNFAIP8 are preferentially expressed in hematopoietic cells^18,19^. We have previously solved the crystal structures of TIPE2 and TIPE3, and found that they possess a unique hydrophobic cavity that is constitutively occupied by phosphoinositides^20,21^. TIPE2 and TIPE3 can bind to lipid second messengers that include phosphatidylinositol 4,5-bisphosphate (PtdIns(4,5)P_2_ or PIP2) and phosphatidylinositol 3,4,5-trisphosphate (PtdIns(3,4,5)P_3_ or PIP3)^22,23^. In addition, TIPE2 can also directly bind and inhibit Rac^24^. TIPE2-deficient myeloid cells are hyper-responsive to Toll-like receptor activation and have enhanced phagocytic and bactericidal activities, and TIPE2-deficient mice are hypersensitive to intravenously induced septic shock^18,24,25^. We report here that TIPE2 and TNFAIP8 play redundant roles in controlling lymphocyte migration. Loss of both TIPE2 and TNFAIP8, but not either alone, is required to stop directional migration of lymphocytes. This Dual Molecular Redundancy (DMR) ensures that the direction of migration is maintained even when one TIPE protein fails (e.g., as a result of gene mutation or downregulation). It enhances the overall robustness of the system, as the DMR does in electric engineering^26^.

## Methods

### Mice

*Tnfaip8*^−/−^ and *Tipe2*^−/−^ C57BL/6 mice were generated as described^18,27^. The *Tnfaip8*^−/−^*Tipe2*^−/−^ double-knockout (DKO) mice were generated by crossing *Tnfaip8*^−/−^ with *Tipe2*^−/−^ mice. WT C57BL/6 mice expressing CD45.1 or CD45.2 and *Rag2*^−/−^ mice were purchased from Jackson Laboratories. Mice were housed in the University of Pennsylvania Animal Care Facilities under pathogen-free conditions. All animal procedures were pre-approved by the Institutional Animal Care and Use Committee of the University of Pennsylvania, and all experiments conform to the relevant regulatory standards.

### Complete blood counts (CBC), and isolation of blood neutrophils (BNs) and splenic CD4^+^ T cells

Blood was drawn from retro-orbital plexus of the eyes. CBC analysis was performed on an automated Hemavet FS950 instrument. BNs were isolated using Histopaque-1119 and Histopaque-1077 (Sigma-Aldrich, St. Louis, MO) according to the manufacturer’s instructions. The purity of BN populations was greater than 70% as determined by flow cytometry after staining with anti-Ly6G-APC (eBioscience, Waltham, MA), and the viability was greater than 90% as determined by trypan blue staining. CD4^+^ T cells were purified from spleens using EasySep Mouse CD4^+^ T Cell Isolation Kit (STEMCELL, Cambridge, MA) or Invitrogen Negative Selection CD4 Purification Kit (Waltham, MA) according to manufacturer’s instructions. The purity of CD4^+^ T cells was greater than 90%, and the viability was greater than 90% as judged by flow cytometry.

### Isolation and enumeration of intestinal IELs

After transcardial perfusion of mice with PBS, Peyer’s patches were removed, and the small intestines were collected and weighed. Intestinal epithelial cells and intraepithelial lymphocytes were stripped by shaking colonic tissue in PBS that contains 5% FBS, 2 mM EDTA, and 1 mM DTT, for 30 min at 37 °C. After filtering through 70-μm cell strainers (BD Biosciences), cells were counted and the total number of cells per gram of intestinal tissue calculated. Cells were then incubated with Fc blockers for 15 min at RT, stained with fluorescent anti-CD45 and anti-CD3 antibodies, as well as with Zombie Aqua Fixable Viability kit (Biolegend), and analyzed by flow cytometry. The total numbers of CD3^+^ and CD45^+^ cells per gram of the small intestine in each mouse were normalized to those of the WT group for each experiment.

### Transmigration assay

CD4^+^ T cells were purified from mouse spleens using Invitrogen negative selection beads (Waltham, MA), stimulated with 1 μg/ml anti-CD3 (Clone 2C11, eBioscience, San Diego, CA) and 1 μg/ml anti-CD28 (Clone 37.15, Biolegend, San Diego, CA) for 2 days, and rested for 3–5 days in a complete RPMI culture medium containing 10% FBS, 1% Glutamine, 1x Pen/Strep, and 10 ng/ml IL-2 (Invitrogen, Frederick, MD). The chemotaxis assay was performed using 96-well Neuro Probe ChemoTx transwell system with 3-μm pore size (Neuro Probe, Gaithersburg, MD) according to manufacturer’s protocol. Briefly, the bottom wells were filled with 30 μl of migration buffer with or without 100 ng/ml CCL21, and CD4^+^ T cells were applied to the upper wells precoated with 15 μg/ml Fibronectin (Sigma, St. Louis, MO). After one hour incubation at 37 °C, migrated cells collected from the bottom wells were quantified using a cell counter. For migration assays with PI3Ks inhibitor (LY294000) and Rac inhibitor (NSC23700), CD4^+^ T cells were pre-incubated with 30 μM LY290004 and 100 μM NSC23766, respectively, for 45 min before the start of the assay. For migration assays with CD4^+^ T cells overexpressing TIPE2 or TIPE2 mutant, CD4^+^ T cells were transfected by electroporation with TIPE2- or TIPE2 mutant-expressing plasmids 24 h before the migration assay.

### Ibidi μ-slide migration assay

Resting splenic CD4^+^ T cells and blood neutrophils were tested in the Ibidi μ-slide migration assay to determine the migration directionality and speed, according to manufacturer’s instructions (Ibidi, Madison, WI)^23^. Briefly, resting WT, *Tnfaip8*^−/−^, *Tipe2*^−/−^, and DKO splenic CD4^+^ T cells or neutrophils as prepared in the Transmigration Assay above were suspended in migration buffer (RPMI medium supplemented with 5% FBS and 1% HEPES), and loaded into Collagen IV-coated Ibidi μ–slides. After resting for 30–45 min at 37 °C, CCL21 (for T cells) or CXCL1 (for neutrophils) was added to one of the reservoirs to a concentration of 200 ng/ml. For measuring the directionality and speed of random migration, cells were prepared as described above but with no chemoattractant added to the chamber. Cells were recorded every 45 sec for at least 2 h using a Leica DMI4000 microscope with Yokogawa CSU-X1 spinning disk confocal attachment at 10× magnification. Images were analyzed by Volocity software (Perkin Elmer, Waltham, MA) using the automated tracking protocol. Objects less than 16 μm^3^ and static were excluded. Tracks less than 50 μm were also excluded. Cell velocity and vector angle between each track’s starting and end points were obtained from Volocity using these settings. Velocity was defined as a cell’s centroid movement in μm/min along the total path. Cell directionality (aka Directional Meandering Index, DMI) was defined as the cosine of the migration angle or the sine of the bearing angle. A value of 1 indicates migration directly towards the chemoattractant, while a value of −1 indicates migration away from the chemoattractant. The tracks were sorted by migration length, and tracks were selected from the middle of the videos (track time was ~60 min).

### PtdIns(3,4,5)P_3_ measurement in live CD4^+^ T cells using biosensors

The PtdIns(3,4,5)P_3_ in live cells was visualized using EGFP-tagged AKT-PH domain, which specifically binds to this phosphoinositide. Briefly, splenic CD4^+^ T cells were stimulated with anti-CD3 and anti-CD28 for 40 h, and then transfected with GFP-C1-AKT-PH vector (Addgene, Watertown, MA) using mouse T cell Nucleofector Kit and Amaxa Nucleofector II (program X-001, Lonza). Cells were cultured for 24 h after the transfection in the presence of 10 ng/ml IL-2. The live CD4^+^ T cells were purified using the Dead Cell Removal Kit (Miltenyi Biotec, San Diego, CA), cultured in fibronectin-coated slide chambers for 45 min, and then stimulated with 600 ng/ml CCL21 applied as a point source from a pipette, in the presence of 5 μM latrunculin A, at 37 °C. The cells were recorded every 10 sec for at least 480 sec using a DeltaVision OMX-SR super-resolution microscope at 60× magnification. The images were analyzed using Volocity and ImageJ softwares. The relative Ptdins(3,4,5)P_3_ level in each cell was calculated as follows: fluorescence intensity of 50% of the cell perimeter that faced the source of the chemokine/fluorescence intensity of 50% of the cell area that faced the source of the chemokine.

### Immunofluorescence confocal microscopy of fixed CD4^+^ T cells

Resting splenic CD4^+^ T cells as prepared in the Transmigration Assay above were subjected to point-source stimulation with CCL21 at 1 μg/ml for 0, 1, and 10 min at 37 °C. Cells were immediately fixed with 3% paraformaldehyde in PBS for 15 min at 37 °C, permeabilized in PBS containing 0.1% Triton X-100 and 3% BSA for 10 min at RT, and blocked with PBS containing 5% normal goat serum and 3% BSA for 1 h at RT. The cells were stained overnight at 4 °C with anti-TIPE2, anti-TNFAIP8 (Proteintech, Rosemont, IL), or anti-Rac1-GTP (NewEast Biosciences, King of Prussia, PA) in 3% BSA, and then for 1 h at RT with secondary anti-rabbit IgG Fab-AlexaFluor 555, or anti-mouse IgM Fab-Alexa Fluor 488 (Thermo Fisher Scientific, Waltham, MA) in 3% BSA. Slides were dried and covered with ProLong Gold with DAPI (Invitrogen, Waltham, MA). Images were acquired on a Zeiss LSM 510 NLO/META and Zeiss LSM 710 confocal microscope and analyzed using LSM Image Browser, Zen lite (Zeiss), and ImageJ software. Up to 120 cells of each type and condition were analyzed. Morphological cell polarization was determined by phase contrast or differential interference microscopy. Unpolarized T cells were of round shape whereas the polarized cells had flat leading-edges at the front (lamellipodia) and contracted uropodia at the rear. The Rac1-GTP polarization index was calculated using ImageJ by manually selecting each cell and then determining the coordinates for the center of the object (X_object_, Y_object_), and comparing that to the center of Rac1 fluorescent signal (X_signal_, Y_signal_), using the following formula that calculated the cellular polarization in terms of cartesian displacement of the center of the object from the center of the signal: $1-|\frac{{X}_{signal}}{{X}_{object}}\ast \frac{{Y}_{signal}}{{Y}_{object}}|$.

### Imaging flow cytometry

The ImageStream (Amnis, Seattle, WA) two camera system with 405, 488 and 658 nm lasers was used for imaging flow cytometry^28^. The system was calibrated using SpeedBead (Amnis, Seattle, WA) prior to use and samples were acquired at optimized laser strength (50–100 mW) with an area classifier (number of pixels in μm^2^) set at 25. Images (TNFAIP8 or TIPE2 in channel 11, nucleus in channel 7, F-actin in channel 2, CD4 in channel 3, and bright field in either channel 1 or 9) were acquired for each cell at 60× magnification and ~20,000 cells were analyzed for each sample, and 2,000 cells were acquired for each compensation control. The integrated software INSPIRE (version 6.0.154, Amnis) was used for data collection. Analysis was performed on the compensated image files using algorithms in IDEAS (version 4.1.146, Amnis) image analysis software. The bright field area versus aspect ratio features were plotted and used to gate on single cells. The gradient root mean square (GRMS) was used to gate on cells that were in focus. The bright field aspect ratio score was plotted against a normalized frequency of cells to generate histograms. Polarized cells were defined as those that had low aspect ratio scores (<0.5) whereas unpolarized cells were those that had high aspect ratio scores (>0.8).

### DNA constructs

Expression plasmids of murine TNFAIP8 isoform 1 and the PH domains from phospholipase C-δ1 (PLCδ-PH) and general receptor for phosphoinositides (GRP1-PH) were constructed by cloning PCR-amplified cDNA into pET-SUMO vector (LifeSensors, Malvern, PA) in frame with the N-terminal 6His-SUMO tag. The TIPE2 Entrance mutant was generated by replacing amino acid residues 28 H, 75 R, 91 R and 183 K with glutamine (Q) using Stratagene QuikChange II Mutagenesis Kit (Agilent, Santa Clara, CA). TIPE2 and Entrance mutant lentiviral expression plasmids were constructed by cloning the open reading frame of each cDNA into the multiple cloning site of pGL-LU-GFP vector (Addgene, Watertown, MA).

### Plasmid transfection and lentivirus infection

Expression constructs were transfected into Lenti-X 293 T cells (Clontech, Mountain View, CA) using Lipofectamine 3000 reagent (Thermo Fisher Scientific, Waltham, MA) following the manufacturer’s protocol. Lentivirus was produced by co-transfecting pGL-LU-TIPE2-GFP, pGL-LU-TIPE2 Entrance mutant-GFP or FUGW expression vectors with 3^rd^ generation lentiviral packaging plasmids (pRSV-Rev, pCMV-VSV-G, and pMDLg/pRRE). Two batches of lentivirus-containing medium were harvested 24 h and 52 h after transfection, followed by filtering through a 0.45 μm Steriflip PVDF membrane (MilliporeSigma, Burlington, MA). Lentivirus was concentrated 100-fold using Lenti-X Concentrator (Clontech, Mountain View, CA) and titrated by RT-qPCR and EGFP-positive cell counts. Lentivirus infection was performed by incubating cells with medium containing the lentivirus at a multiplicity of infection (MOI) of 20 in the presence of 4 μg/ml Polybrene (Sigma, St. Louis, MO) for 12–24 h. Cells were allowed to recover in complete medium for 24 h and then isolated by fluorescence activated cell sorting (FACS) before being used for experiments.

### Phosphoinositide binding assay

Sedimentation-based phosphoinositide binding assays were performed as previously described^22^. Dioleoylphosphatidylcholine (DOPC) and brominated distearoyl PC (brominated PC) were purchased from Avanti Polar Lipids (Alabaster, AL). PtdIns(4,5)P_2_ and PtdIns(3,4,5)P_3_ were purchased from CellSignals (Columbus, OH). Purified trypsin inhibitor of *Glycine max* (Sigma-Aldrich, St. Louis, MO) was used as a control protein. The following small unilamellar vesicles (SUVs) were used at a concentration of 2 mM (1 mM available lipids for binding): (i) 10% PtdIns(4,5)P_2_ + 10% DOPC + 80% brominated PC, (ii) 10% PtdIns(3,4,5)P_3_ + 10% DOPC + 80% brominated PC, or (iii) 20% DOPC + 80% brominated PC. Proteins were used at a concentration of 5 μM. Samples were incubated 1 h at RT, and subjected to ultracentrifugation as described^22^. The relative amounts of proteins in supernatants and pellets were determined by Coomassie Blue G-250 staining of SDS-PAGE gels containing the resolved proteins.

### Surface plasmon resonance (SPR) assay

Recombinant TNFAIP8, PLCδ-PH and GRP1-PH were expressed from *Escherichia coli* BL21(DE3) cells (Agilent, Santa Clara, CA) and purified using Ni-NTA Agarose (Qiagen, Germantown, MD). 6His-SUMO tagged proteins were eluted with 250 mM Imidazole from beads, followed by cleavage with SUMO Protease 1. The SUMO fusion proteins and SUMO Protease after cleavage were removed by affinity chromatography on a second Ni-chelating resin. Final eluates with untagged native proteins were concentrated using Amicon Ultra centrifugal filters (MilliporeSigma, Burlington, MA), and dialyzed in HBS (25 mM HEPES, 150 mM NaCl, pH 7.4) buffer using Slide-A-Lyzer cassettes (Thermo Fisher Scientific, Waltham, MA). The purified proteins were at least 95% pure as judged from overloaded Coomassie Blue G-250 stained SDS gels. Protein concentrations were determined based on absorbance at 280 nm using calculated extinction coefficients. SPR assays were carried out using a BIAcore T200 instrument (GE Healthcare, Marlborough, MA) measuring PtdIns(4,5)P_2_ and PtdIns(3,4,5)P_3_ binding. Briefly, the surface of L1 sensor chip was cleaned by a 5 min injection of 40 mM octyl D-glucoside at a flow rate of 5 μl/min. Vesicles containing DOPC alone, 3% or 10% (mole/mole) of PtdIns(4,5)P_2_ or PtdIns(3,4,5)P_3_ in a DOPC background that were generated through a 50 nm NanoSizer Liposome Extruder (T&T Scientific, Knoxville, TN), were immobilized on L1 sensor chip surfaces, resulting in signals of around 6500 to 8500 resonance units. Purified test proteins were injected over the surfaces at five or more different concentrations with sequential dilutions, at a flow rate of 3 μl/min. The experiments were all performed at 25^◦^C in HBS buffer (pH 7.4). The SPR signals were detected during the association and disassociation, and the sensorgrams were analyzed using BIAevaluation software. SPR signals were corrected for background (DOPC) binding, and a binding isotherm was generated from equilibrium response (R_eq_) versus the concentration (C) of proteins. The equilibrium dissociation constant (*K*_D_) was derived from steady-state affinity analysis by nonlinear least-squares fitting of the binding isotherm using the equation R_eq_ = R_max_/(1+*K*_D_/C). The percent of maximal binding was determined at each protein concentration as equilibrium response divided by the maximum response measured at saturation.

### PI3K enzymatic assay

PI3K-mediated phosphorylation of PtdIns(4,5)P_2_ was measured by determining ATP consumption using the ADP-Glo Lipid Kinase Assay Kit (Promega, Madison, WI) according to the manufacturer’s instructions. Reaction buffer consisted of 0.4 nM PI3K (p110α/p85α), 50 μM phosphatidylserine + PtdIns(4,5)P_2_ (at 3:1 ratio), 25 μM ATP, and different concentrations of TNFAIP8 protein. 2 μM BSA was used as a negative control protein. Control experiment without phospholipid substrates showed only background levels of ATP consumption. PI3K-catalyzed generation of PtdIns(3,4,5)P_3_ was determined at 60 min after reaction initiation, and the value in the absence of TNFAIP8 protein was set to 1.

### Experimental autoimmune encephalomyelitis (EAE)

The induction and clinical scoring of EAE in mice were performed as we described previously^29,30^. Spinal cords of mice were harvested at the end of each experiment, fixed, paraffin-embedded, and sectioned. The sections were stained with hematoxylin and eosin, and analyzed using a wide field light microscope.

### Bone marrow chimeric experiments

Bone marrow chimeric mice were generated as we described^30^. Briefly, WT or DKO mice were sub-lethally irradiated, and injected intravenously with bone marrow cells from DKO or WT mice (10^7^ cells/mouse). For the mixed bone marrow chimeric experiment, bone morrow cells from CD45.1 WT and CD45.2 DKO mice were mixed at 1:1 ratio and injected into sub-lethally irradiated CD45.1 WT recipient mice. Seven weeks after bone marrow cell transfer, EAE was induced with myelin oligodendrocyte glycoprotein (MOG) 35–55 peptide, and scored on a scale of 0–5 as we described^30^.

### Adoptive transfer of EAE

Donor mice were immunized with MOG peptide^30^ and splenocytes were collected 7 days later. After stimulation with 20 μg/ml MOG peptide for three days in culture and removal of dead cells, the splenocytes were subjected to negative selection using EasySep Mouse CD4^+^ T Cell Isolation Kit (STEMCELL, Cambridge, MA). The purified CD4^+^ cells were injected to *Rag2*^−/−^ recipient mice (2–3 × 10^6^ cells/mouse) intravenously. Pertussis toxin (100 ng/mouse) was injected intraperitoneally on the day of the cell transfer and the day after. EAE was scored on a scale of 0–5 as we described^30^.

### Leukocyte tracking in mice

WT and *Tnfaip8*^−/−^*Tipe2*^−/−^ KO mice were immunized with MOG35-55 peptide as we described^30^. Two weeks later, splenocytes were harvested and stimulated with MOG35-55 peptide (20 μg/ml) for 3 days. Live WT and KO cells were then labeled with fluorochromes CMTMR and CMFDA, respectively, mixed at 1:1 ratio, and injected via tail vein (1 million cells/mouse) into B6 mice that had been immunized for EAE ten days earlier with MOG. Mice were sacrificed on the day of EAE onset, and their blood, spleens, and spinal cords collected. The percentages of transferred WT and KO cells among total leukocytes isolated from each sample were determined by flow cytometry.

## Results

### Complete loss of the directionality of leukocytes deficient in TNFAIP8 and TIPE2

**Figure 1:**
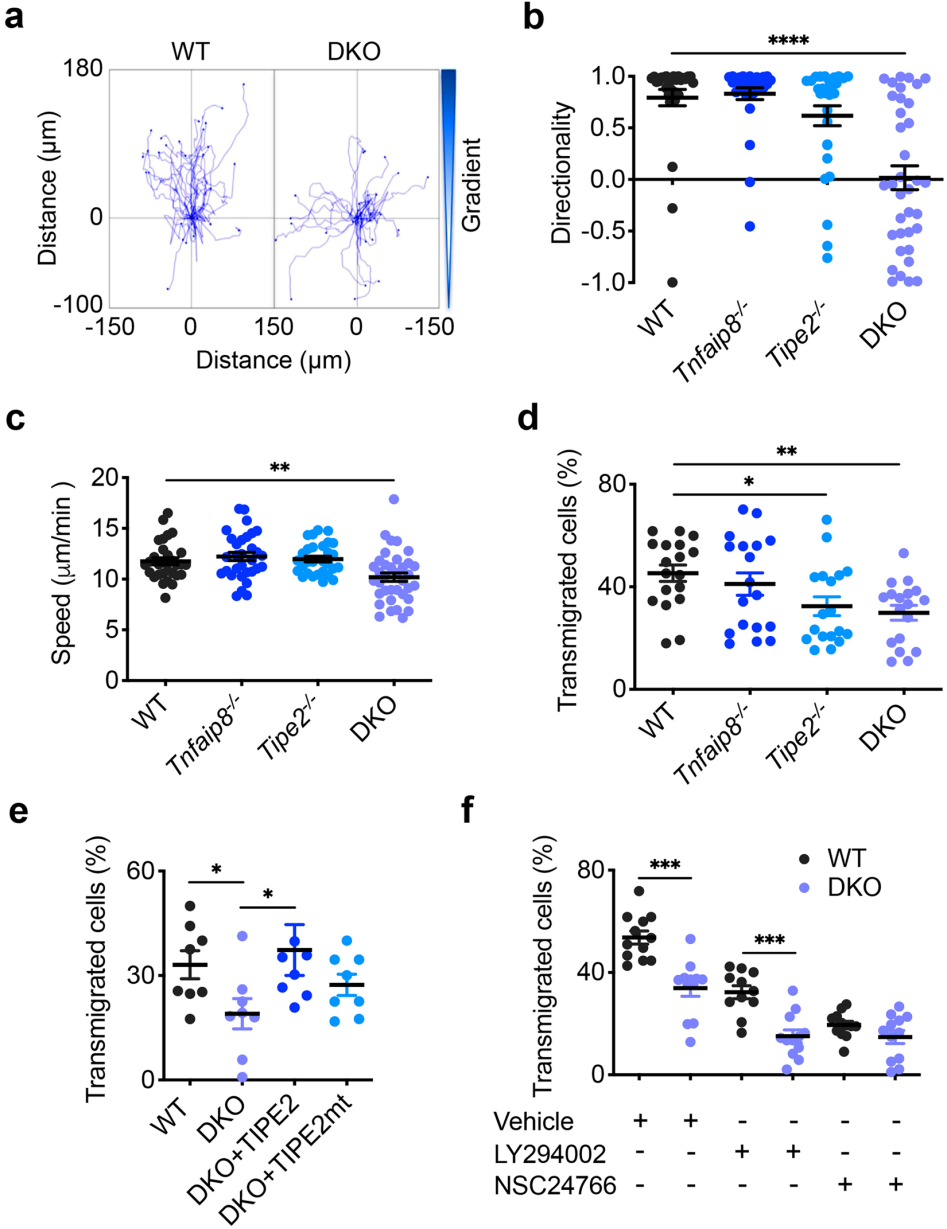
Complete loss of directionality, but not speed, of TIPE-deficient T cells. (**a–c)** Migration tracks (**a**) directionality (**b**) and speed (**c**) of CD4^+^ T cells from WT, *Tnfaip8*^−/−^, *Tipe2*^−/−^, and *Tnfaip8*^−/−^*Tipe2*^−/−^ (DKO) mice (4 mice per group), in response to CCL21, as determined in the μ-slide migration assay. n = 25 cells per group (**a**); n = 30 cells for WT, 31 for *Tnfaip8*^−/−^, 29 for *Tipe2*^−/−^ and 36 for DKO group (**b,c**). (**d**–**f)** Transmigration of CD4^+^ T cells from mice of the indicated genotypes (4 mice per group) (**d**, n = 18 samples per group), together with DKO cells transfected with the expression plasmids for TIPE2 or TIPE2 mutant (TIPE2mt) (**e**, n = 8 per group), or cells treated with the PI3K inhibitor LY290004 or Rac inhibitor NSC23766 (**f**, n = 12 per group), as determined in the transwell transmigration assay with CCL21. Data are representative of at least three independent experiments (**a-c**), or are pooled from three (**e**,**f**) or four experiments (**d**). Values are mean ± s.e.m. of n biologically independent samples (**b**–**f**). *P < 0.05; **P < 0.01; ***P < 0.001; ****P < 0.0001 (Mann-Whitney *U* test (**b**) or Student’s *t*-test (**c**–**f**)).

To determine the potential roles of TIPE2 and TNFAIP8 in T lymphocyte migration, we isolated splenic CD4^+^ T cells from wild-type (WT), *Tipe2*^–/–^, *Tnfaip8*^–/–^, and *Tipe2*^–/–^*Tnfaip8*^–/–^ (double knockout, or DKO) C57BL/6 mice, and studied them in both μ−slide (for two-dimensional migration) and transwell chamber (for transmigration) assays. We found that TIPE2 and TNFAIP8 deficiency completely abolished the directionality of T cells during chemokine-induced migration (chemotaxis) (Fig. 1). Specifically, in the μ-slide assay, time-lapse video microscopy that tracked the trajectory of individual migrating cells following treatment with chemokine CCL21 revealed that the WT T cells had a directionality of 0.79 ± 0.08 (with 1.00 being the highest value of directionality); this was reduced to 0.02 ± 0.12 for the DKO T cells (Fig. 1a,b, Supplementary Videos 1, 2). WT T cells traveled with an average velocity of ~12 μm/minute, which was only slightly reduced for DKO T cells (Fig. 1c). Notably, WT and DKO T cells not treated with chemokines migrated randomly with much-reduced speed (6.89 μm/minute for WT and 6.60 μm/minute for DKO group) and directionality (Supplementary Fig. 1a,b), indicating that chemokines could increase migration speed of both WT and KO T cells through chemokinesis. Importantly, deficiency in either TIPE2 or TNFAIP8 alone did not significantly affect either directionality or velocity. These results indicate that TIPE2 and TNFAIP8 play redundant roles in controlling T cell directionality, and loss of one can be adequately compensated by the other.

Similarly, in the transwell assay, DKO T cells exhibited a severe defect in transmigration, although TIPE2 deficiency alone, but not that of TNFAIP8, also significantly reduced T cell transmigration (Fig. 1d). The migration defect of DKO T cells could be completely rescued by re-expressing TIPE2, but not by expressing a TIPE2 mutant that had reduced ability to bind PtdIns(4,5)P_2_ (the TIPE2 mutant had its 28 H, 75 R, 91 R, and 183 K amino acid residues replaced by Q) (Fig. 1e). The transmigration of both WT and DKO T cells could be reduced by inhibitors for either PI3-kinases (PI3Ks) or Rac, but the difference between WT and DKO T cells disappeared only in the presence of the Rac inhibitor (Fig. 1f), suggesting that Rac is important for TIPE regulation of T cell migration. By flow cytometry, we found that the majority of migrating cells in this assay were central memory T cells (CD4^+^CD44^high^CD62L^−^).

Notably, neither TIPE2 nor TNFAIP8 deficiency jeopardized random migration, as transmigration of single or double KO T cells in the absence of chemokines were either normal or slightly increased as compared to WT T cells (Supplementary Fig. 1c). Furthermore, chemokine CCL21 appeared to increase random transmigration of both WT and KO T cells (presumably through chemokinesis), since the chemokine-induced transmigration (Fig. 1d) was calculated by subtracting random migration (Supplementary Fig. 1c) from total migration.

The effect of TIPE2 and TNFAIP8 on T cell migration was not limited to CCL21. CXCL12-induced migration of CD4^+^ T cells was also significantly reduced in DKO group (Supplementary Fig. 1d). Additionally, the critical roles of TIPE2 and TNFAIP8 in establishing cell directionality were not restricted only to T cells. We previously reported that TIPE2 deficiency in murine neutrophils partially affected their chemotaxis^23^. Here we extended that observation to TNFAIP8 and found that neutrophils deficient in both TIPE2 and TNFAIP8 completely lost their directionality (from 0.62 ± 0.08 in the WT to 0.04 ± 0.04 in the DKO group), while preserving relatively high migrating speed in response to chemokine CXCL1 (Supplementary Fig. 1e,f, Supplementary Videos 3, 4). Thus, TIPE proteins selectively control the direction of migration with minimal effects on the migration speed of leukocytes.

### TNFAIP8 and TIPE2 pilot lymphocytes through PI3Ks and Rac

**Figure 2:**
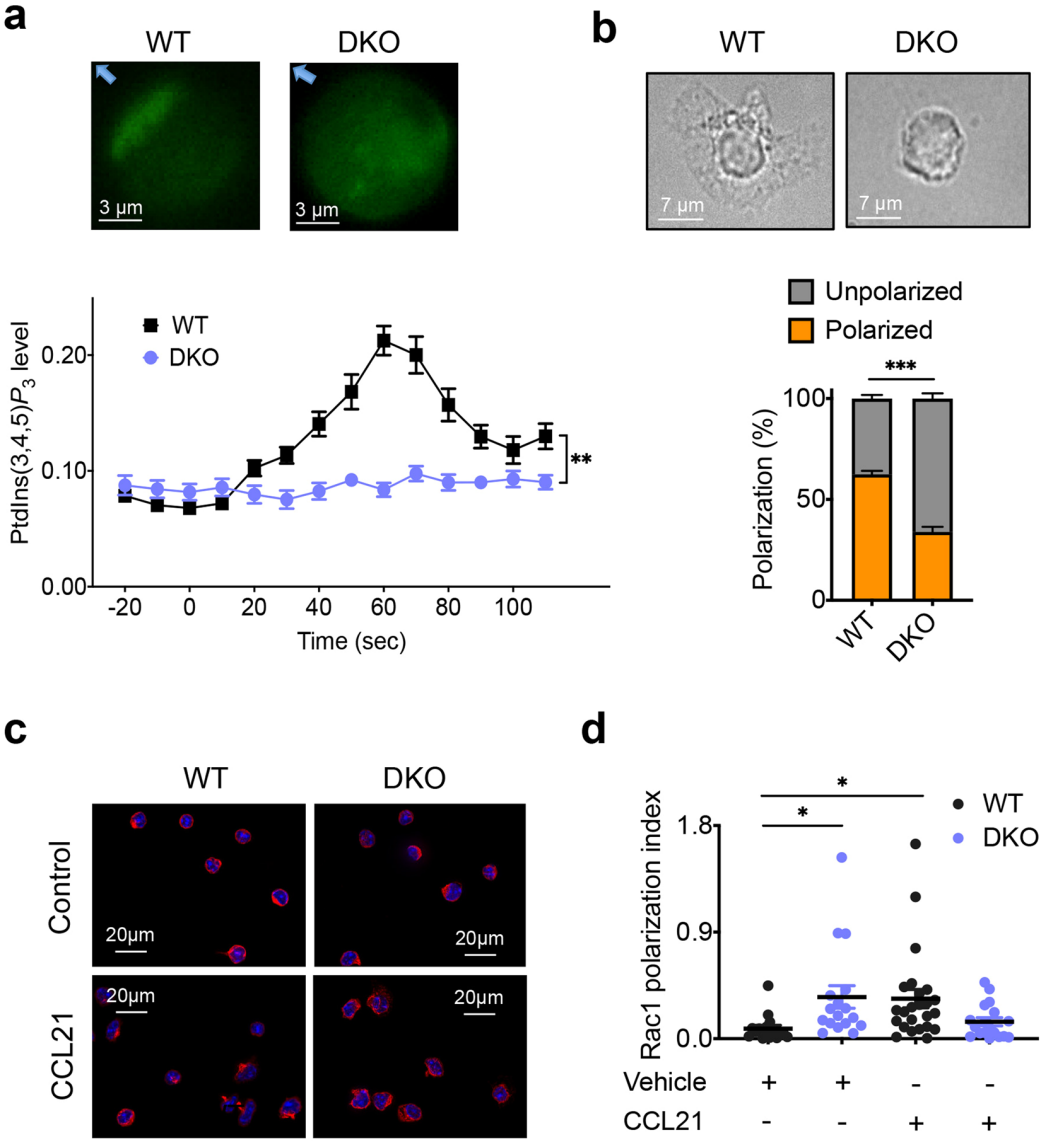
Essential roles of TIPE proteins in chemokine-induced PtdIns(3,4,5)P_3_ generation, and morphological and Rac1-GTP polarization. (**a**) PtdIns(3,4,5)P_3_ levels detected by AKT-PH-GFP biosensor in live WT and DKO CD4^+^ T cells, in response to point source stimulation of CCL21, as visualized by super-resolution fluorescence video microscopy. Upper panels show a WT and a DKO T cells 60 sec after CCL21 stimulation, with arrows pointing to the source of CCL21. The lower panel shows the changes in PtdIns(3,4,5)P_3_ levels relative to Time 0 when CCL21 was applied; values are mean ± s.e.m., with n = 20 cells for each group. (**b**) Morphological polarization of WT and DKO CD4^+^ T cells, in response to point source stimulation of CCL21, as visualized by phase contrast microscopy. Upper panels show a polarized WT and an unpolarized DKO T cells 10 min after CCL21 stimulation. The lower panel shows the percentages of polarized and unpolarized cells 10 min after CCL21 stimulation; n = 308 cells for WT and 304 cells for DKO group. (**c,d)** Rac1-GTP polarization in WT and DKO CD4^+^ T cells, in response to point source stimulation of CCL21, as visualized by immunofluorescence microscopy. Panel-c shows WT and DKO T cells 60 sec after incubation with CCL21 or medium alone (*Control*). Panel-d shows the calculated polarization index of each group treated as in Panel-c; n = 16–24 cells for WT and 17–18 for DKO group. Values are mean ± s.e.m. The experiments were repeated independently at least three times (**a**–**d**) with similar results. *P < 0.05; **P < 0.01; ****P < 0.0001 (Mann-Whitney *U* test (**a**) or Student’s *t*-test (**b**,**d**)).

To explore how TNFAIP8 and TIPE2 control cell directionality, we studied PI3Ks and Rac signals, as well as morphological polarization of T cells. PI3Ks signaling was measured in live T cells using a PtdIns(3,4,5)P_3_-specific biosensor, i.e., the enhanced green fluorescent protein (eGFP)-tagged AKT-PH domain. By time-lapse video microscopy, we compared PtdIns(3,4,5)P_3_ generation in WT and DKO T cells in response to point-source CCL21 stimulation over a period of 110 seconds (Fig. 2a). CCL21-induced PtdIns(3,4,5)P_3_ production occurred immediately after chemokine stimulation, at the side of the cell that faced the chemokine source, reaching its peak level ~60 seconds later. By contrast, no significant increases in PtdIns(3,4,5)P_3_ were observed in DKO T cells. Consistent with these observations, chemokine-induced morphological polarization was significantly reduced in DKO T cells as compared to WT T cells (Fig. 2b). TIPE2 has been reported to be a global inhibitor of Rac^23,24^. In support of this view, Rac-GTP polarization occurred constitutively in DKO T cells, which was not further increased by chemokine treatment (Fig. 2c,d). By contrast, WT T cells responded to the chemokine treatment by polarizing their Rac-GTP. These results indicate that TIPE proteins are essential for generating the phosphoinositide and Rac signals required for steering cells toward chemoattractants.

TIPE2 and TNFAIP8 were present in most parts of the cytosol and plasma membrane in unpolarized T cells. By contrast, in polarized T cells, both TIPE2 and TNFAIP8 moved to the leading edges of the cells, overlapping with F-actin (Supplementary Fig. 2a,b). During the CCL21-induced chemotaxis, leading edges of WT T cells were generated mostly by pseudopod splitting, consistent with reports from others for granulocytes and *Dictyostelium discoideum* cells^31^. In contrast, DKO T cells generated their leading edges by de novo pseudopod formation as frequently as pseudopod splitting (Supplementary Fig. 2c), indicating that the latter cells might have lost the ability to follow chemokine gradient.

### TNFAIP8 is a carrier protein of phosphoinositide second messengers, and an enhancer of PI3Ks

**Figure 3:**
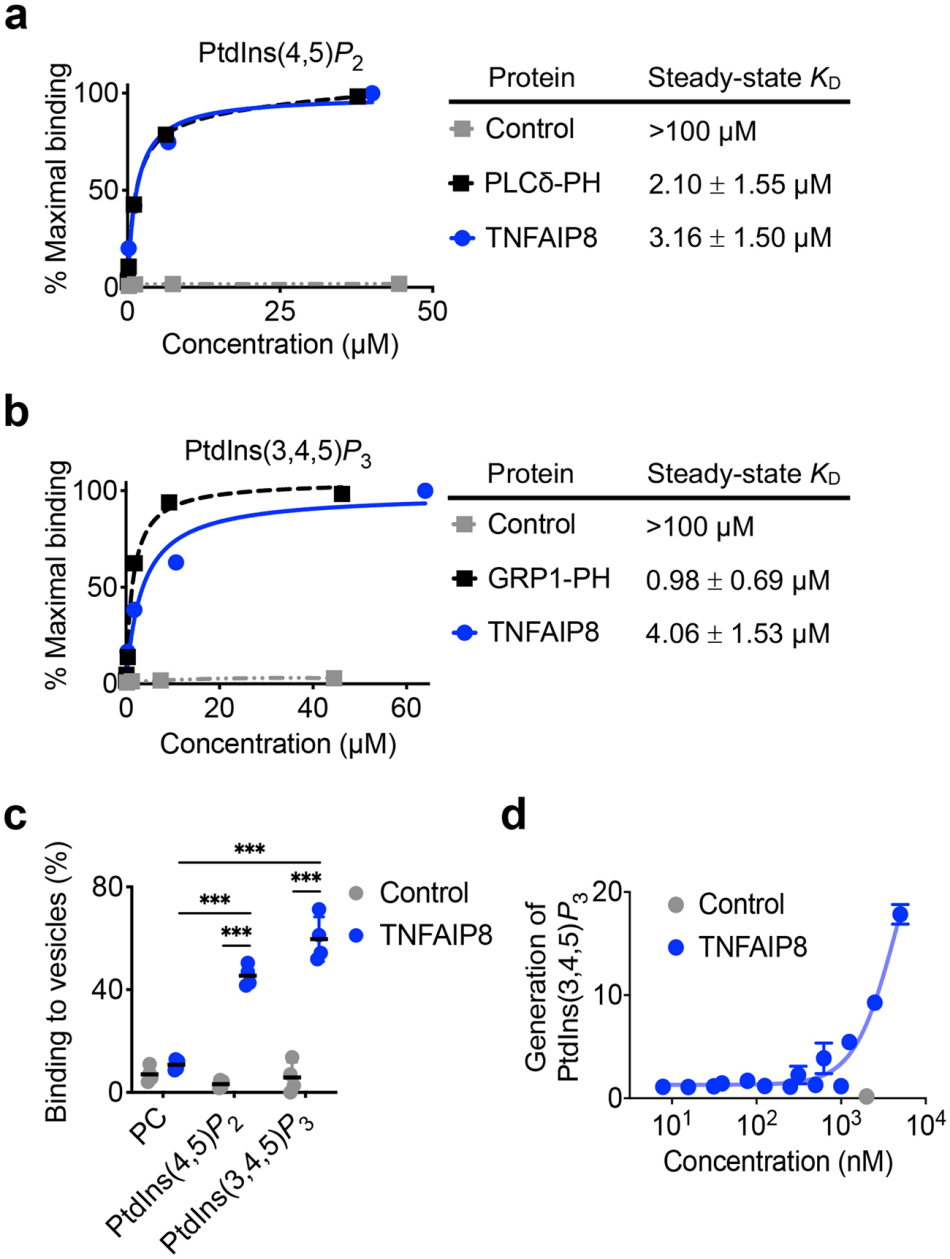
TNFAIP8 binding to phosphoinositides, and its effect on PtdIns(3,4,5)P_3_ generation by PI3Ks. (**a,b**) SPR analysis of TNFAIP8 binding to DOPC membranes containing 10% (mole/mole) PtdIns(4,5)P_2_ (**a**) or PtdIns(3,4,5)P_3_ (**b**) on L1 sensor chip. Purified PLCδ-PH and GRP1-PH domains were used as positive controls, and trypsin inhibitor as a negative control (*Control*). The percent of maximal binding (Left Panels) and equilibrium *K*_D_ (Right Panels) are shown. (**c**) Sedimentation-based phosphoinositide binding assay showing the proportion of TNFAIP8 and control protein trypsin inhibitor bound to SUVs containing 100% PC, 10% PtdIns(4,5)P_2_ or 10% PtdIns(3,4,5)P_3_. (**d**) Phosphorylation of PtdIns(4,5)P_2_ by PI3Ks as measured in the ADP-Glo kinase assay, in the presence of increasing concentrations of murine TNFAIP8 protein or 2 μM BSA (*Control*). The values are mean ± s.d. (**a–c**) or mean ± s.e.m. (**d**). Data represent three independent experiments (**a,b**) or are pooled from four (**c**) or two independent experiments done in duplicates (**d**). ***P < 0.001 (Student’s *t*-test (**c**)).

As we recently reported, TIPE2 is a carrier protein of both PtdIns(4,5)P_2_ and PtdIns(3,4,5)P_3_^23^, but the affinity of TIPE2 to these lipid second messengers are unknown. To determine whether TNFAIP8 is also able to bind to these lipids, we performed surface plasmon resonance (SPR) and vesicle sedimentation assays, and found that TNFAIP8 bound PtdIns(4,5)P_2_ and PtdIns(3,4,5)P_3_ in both assays (Fig. 3a–c). The steady-state equilibrium dissociation constants (*K*_D_) of TNFAIP8 to PtdIns(4,5)P_2_ and PtdIns(3,4,5)P_3_ were 3.16 and 4.06 μM, respectively (Fig. 3a,b), suggesting that TNFAIP8 bound to these lipids with medium affinities.

As we reported, TIPE2 could act as an “enhancer” for PI3Ks^23^. To determine whether TNFAIP8 could also promote the activity of PI3Ks, we performed an ADP-Glo PI3K assay. Addition of TNFAIP8 to the PI3K assay significantly potentiated PtdIns(3,4,5)P_3_ generation in a dose-dependent manner (Fig. 3d). These results indicate that the reduced PtdIns(3,4,5)P_3_ generation in DKO T cells (Fig. 2a) was likely due to the loss of the enhancer function of both TNFAIP8 and TIPE2.

### Abnormal positioning of leukocytes in mice deficient in TNFAIP8 and TIPE2 under steady state

**Figure 4:**
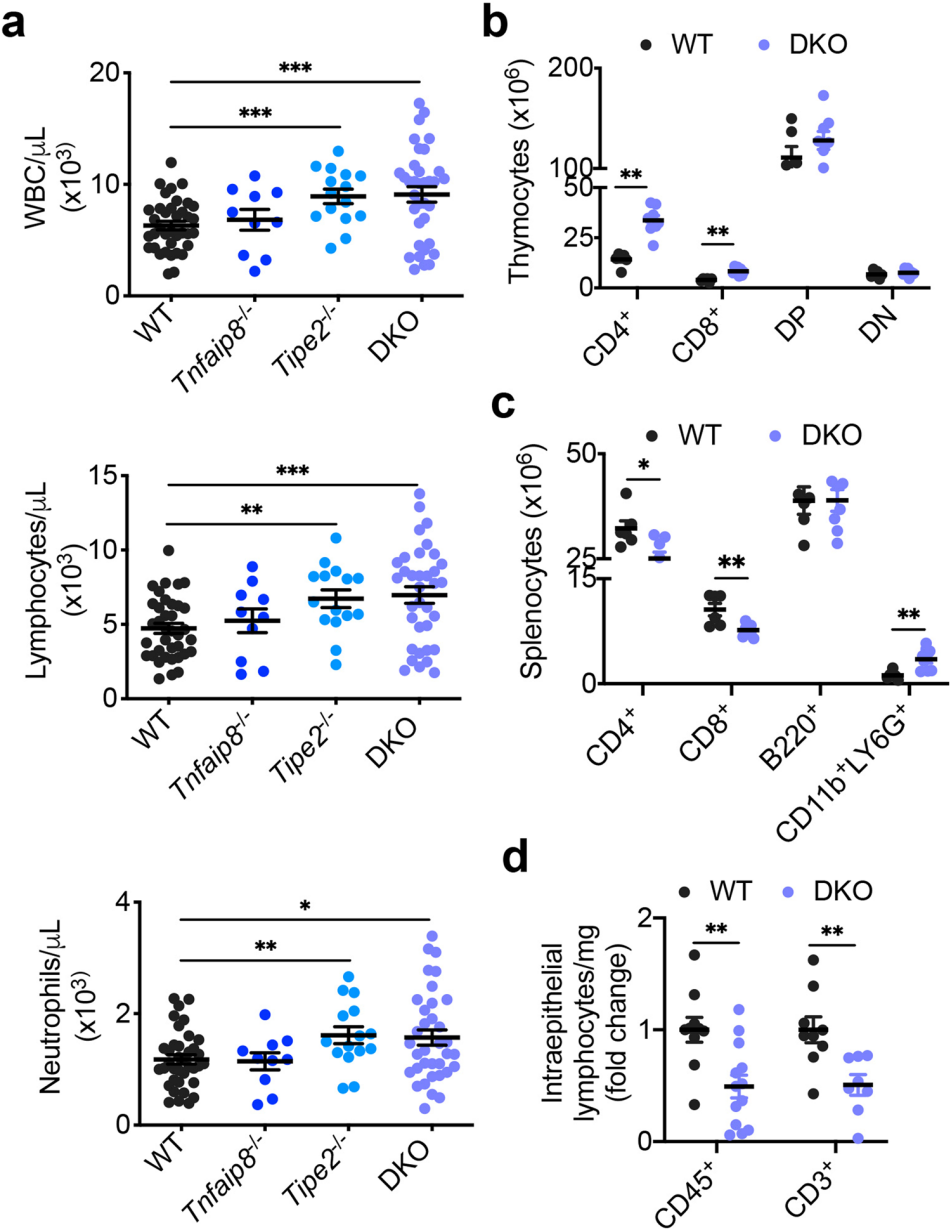
Abnormal positioning of leukocytes in tissues of TIPE-deficient mice under the steady state. (**a**) The total white blood cell, lymphocyte, and neutrophil counts of the blood of normal 6–8-week-old WT (n = 37), *Tnfaip8*^−/−^ (n = 10), *Tipe2*^−/−^ (n = 15), and DKO (n = 35) mice. (**b**) The numbers of the indicated cell subsets of thymus of WT (n = 6) and DKO (n = 8) mice as determined by flow cytometry. CD4^+^ and CD8^+^ denote the respective single positive cells; DP denotes double positive cells; DN denotes double negative cells. (**c)** The numbers of the indicated cell subsets of spleen of WT (n = 6) and DKO (n = 8) mice as determined by flow cytometry. (**d)** Relative numbers of the indicated intraepithelial cells per mg of small intestine of WT (n = 10 mice for CD45^+^ and 9 for CD3^+^ cells) and DKO (n = 13 for CD45^+^ and 8 for CD3^+^ cells) mice as determined by flow cytometry. Data are normalized to the mean of the respective WT group. The values are mean ± s.e.m. (**a–d**), and are pooled from two (**b,c**) or three (**a,d**) independent experiments. *P < 0.05; **P < 0.01; ***P < 0.001 (Student’s *t*-test (**a–d**)).

Lymphocytes are deployed strategically throughout the body via two distinct modes of migration: directed and random migrations^9,32^. The degree to which each mode of migration contributes to the deployment of lymphocytes in each tissue is not well understood. Chemokines enhance not only directed migration but also random migration (Fig. 1)^32,33^. The selective defect in directed, but not random, migration of leukocytes deficient in TIPE2 and TNFAIP8 allowed us to examine the contribution of directed migration to tissue-specific deployment of leukocytes in mice. Deficiency in TIPE2 and TNFAIP8 significantly increased the total numbers of white blood cells (WBC) in the blood, which included both lymphocytes and neutrophils (Fig. 4a). Deficiency in TIPE2 alone, but not in TNFAIP8, also significantly increased blood leukocyte numbers. The total CD4^+^CD8^-^ and CD8^+^CD4^-^ T cells in the thymus were also significantly increased in DKO mice (by ~2 fold)(Fig. 4b). By contrast, the total CD4^+^CD8^-^ and CD8^+^CD4^-^ T cells in the spleen were significantly decreased in DKO mice (Fig. 4c), suggesting a defect in T cell migration from thymus to spleen. The numbers of other leukocyte subsets except that of CD11b^+^Ly6G^+^ cells in the spleen were not significantly affected by TIPE2 and TNFAIP8 deficiency. The weights of, and total numbers of cells in, lymphoid organs, which included thymus, spleen, and mesenteric lymph node, were not significantly affected by TIPE2 and TNFAIP8 deficiency (Supplementary Fig. 3). This is consistent with our previous report that C57BL/6 mice deficient in either TIPE2 or TNFAIP8 alone do not have significant changes in their total lymphocyte numbers in lymphoid organs under the steady state (before 10 weeks of age)^24,27^. These results indicate that, under steady state, directed migration is critical for T cell deployment to lymphoid organs, and that random migration (which can be significantly enhanced by chemokines) also plays an important role in leukocyte deployment to lymphoid organs.

Under steady-state, ~50% of T lymphocytes in the body are deployed to the mucosal epithelium^34^. How intraepithelial lymphocytes (IELs) move into the mucosal epithelium is not well understood. Remarkably, the total numbers of CD45^+^ and CD3^+^ IELs in the intestinal epithelium decreased by ~50% in DKO mice (Fig. 4d). This indicates that about half of the IELs move to the intestinal epithelium through TIPE-dependent migration.

### Resistance of mice deficient in TNFAIP8 and TIPE2 to autoimmune encephalomyelitis

Under inflammatory conditions, lymphocytes can exit the blood circulation and enter non-lymphoid tissues. This is crucial for the development of inflammatory diseases such as multiple sclerosis (MS)^5,6^. To determine the roles of TIPE2 and TNFAIP8 in experimental autoimmune encephalomyelitis (EAE), an animal model for MS, we immunized WT, *Tipe2*^–/–^, *Tnfaip8*^–/–^, and *Tipe2*^–/–^*Tnfaip8*^–/–^DKO mice with myelin oligodendrocyte glycoprotein (MOG) peptide 35–55, and monitored daily for clinical signs of EAE (Supplementary Fig. 4a). We found that the onset and mean EAE scores were significantly reduced in all knockout groups with the DKO group being the most affected. This indicates that TIPE2 and TNFAIP8 play redundant roles in EAE, and loss of one is significantly compensated by the other. The fatality was reduced from 60% in the WT group to 0% in all KO groups. Consistent with these clinical findings, histological examination of spinal cord sections revealed significant differences in the degree of leukocyte infiltration between WT and KO groups (Supplementary Fig. 4b,c). In the WT group, multiple inflammatory foci were observed, with extensive leukocyte infiltration into the white matter. By contrast, leukocyte infiltration in KO mouse spinal cords was much less pronounced.

To define the roles of TIPE2 and TNFAIP8 expressed by hematopoietic cells, we studied EAE in irradiated WT and DKO female C57BL/6 mice that had received bone marrow from either WT or DKO mice 7 weeks earlier (Supplementary Fig. 4d). In the chimeric mice, ~90% of leukocytes were derived from donor bone marrow as determined by flow cytometry. Notably, following immunization with MOG peptide, mice that received DKO bone marrow developed significantly less EAE than those reconstituted with WT cells. Therefore, loss of TIPE2 and TNFAIP8 in hematopoietic cells alone is sufficient to significantly hinder the development of EAE. Because not all hematopoietic cells in recipient mice can be eliminated by irradiation, whether and to what degree TIPE proteins expressed by non-hematopoietic cells contribute to EAE cannot be conclusively established in this model.

**Figure 5:**
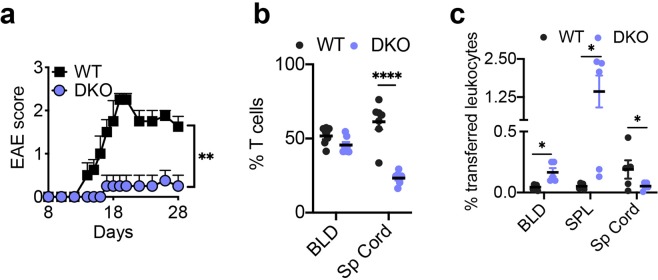
Abnormal positioning of leukocytes in the nervous tissue of TIPE-deficient mice during encephalomyelitis. (**a**) EAE scores of *Rag2*^−/−^ mice (n = 4 mice per group) that were injected with MOG-specific WT or DKO CD4^+^ T cells. (**b)** The percentages of WT CD45.1^+^ and DKO CD45.2^+^ T cells among total CD3^+^ T cells in the blood (*BLD*) and spinal cord (*Sp Cord)* of EAE mice (n = 7 mice per group) that were injected with WT and DKO bone marrow cells at 1:1 ratio. Samples were collected one day after EAE onset. (**c**) The percentages of transferred CMTMR-labeled WT and CMFDA-labeled DKO cells among total leukocytes in the blood (*BLD*), spleen (*SPL*), and spinal cord (*Sp Cord)* of EAE mice (n = 5 mice per group) that were injected with the respective MOG-specific WT and DKO CD4^+^ T cells at 1:1 ratio. Samples were collected on the day of the EAE onset. The values are mean ± s.e.m. (**a–c**), and are pooled from two independent experiments (**b,c**) or are representative of four independent experiments (**a**). *P < 0.05; **P < 0.01; ****P < 0.0001 (Mann-Whitney *U* test (**a,b**) or Student’s *t*-test (**c**)).

MOG-induced EAE is a T cell-initiated disease. To determine whether TIPE deficiency in T cell alone is sufficient to affect EAE, we adoptively transferred activated anti-MOG T cells from WT and DKO mice into Rag2-deficient B6 mice (that had endogenous myeloid but not lymphoid cells). We found that EAE was significantly diminished in mice received DKO T cells (Fig. 5a). To measure anti-MOG responses of T cells, mice were sacrificed 10 days after immunization, and their splenocytes cultured in the presence of the MOG peptide. DKO cell cultures produced increased interleukin (IL)-2 and IL-17A as compared to WT cultures (Supplementary Fig. 4e,f). Because the frequency of MOG-specific T cells in different groups could be different, we also compared responses of purified CD4^+^ T cells from naïve mice to anti-CD3 and anti-CD28 stimulation *in vitro*. Similar to its effect on anti-MOG responses, TIPE2 and TNFAIP8 double deficiency increased T cell responses to anti-CD3 and anti-CD28 stimulation (unpublished data). These results indicate that reduced EAE in TIPE-deficient mice is not due to reduced T cell responses to MOG.

### Abnormal positioning of leukocytes in the central nervous system of mice deficient in TNFAIP8 and TIPE2 during autoimmune encephalomyelitis

Reduced EAE in TIPE-deficient mice could be due to reduced migration of KO T cells into the central nervous system (CNS), to which access of leukocytes is tightly controlled by the blood-brain barriers^35,36^. In the mixed bone marrow chimeric mice that had both CD45.1 WT and CD45.2 DKO T cells, markedly reduced numbers of DKO T cells were found in the CNS as compared to WT T cells, following the onset of EAE (Fig. 5b). Similarly, upon co-transfer into mice with EAE, CMTMR-labeled DKO cells showed a marked defect in infiltrating CNS, as compared to CMFDA-labeled WT cells in the same mice (Fig. 5c). This was accompanied by a significant increase of the transferred DKO cells in the blood and spleen of the mice. These results indicate that T cells deficient in TNFAIP8 and TIPE2 have a severe defect in infiltrating the CNS during neural inflammation.

## Discussion

**Figure 6:**
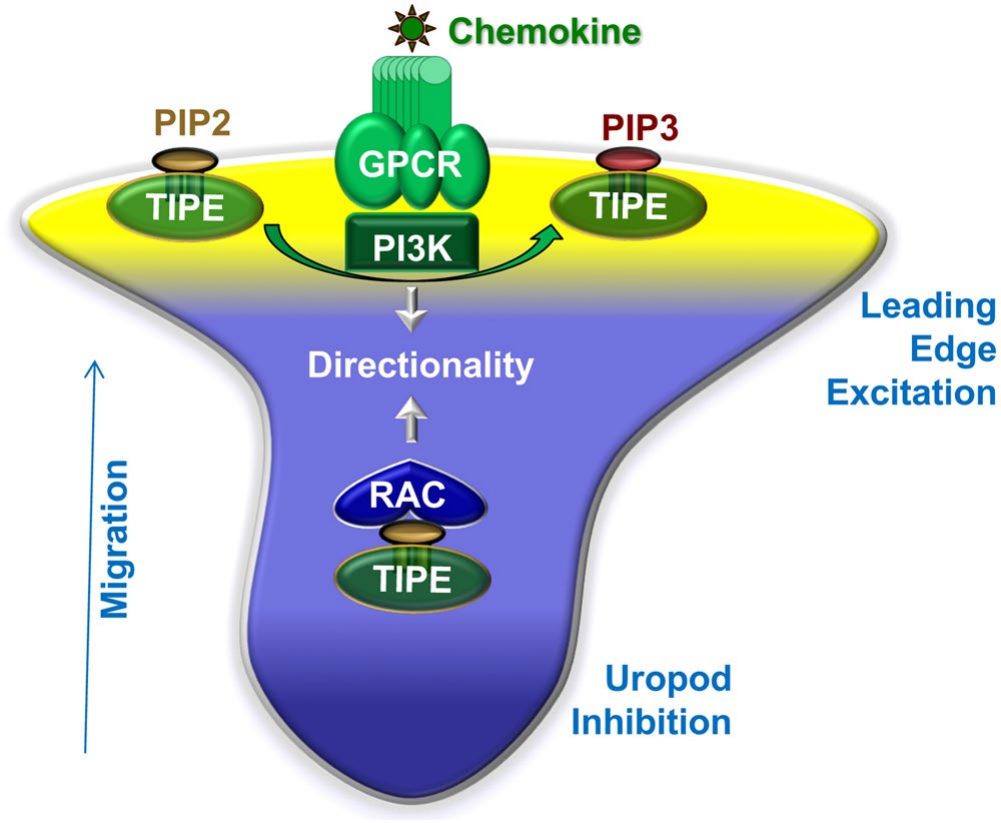
The TIPE molecular pilot. Phospholipid second messengers are present in two distinct forms in cells: the lipid membrane-anchored form and the TIPE-anchored form, e.g., TIPE-PIP2 and TIPE-PIP3. The membrane-anchored form has its acyl chains inserted into the lipid bilayer and can function only at the membrane. On the other hand, the TIPE-anchored form of phosphoinositides has its acyl chains hidden inside the TIPE hydrophobic cavity and its charged head group exposed on the surface. The TIPE-anchored form is water-soluble and freely diffusible, and can function at the membrane-cytosol interface and in the cytosol. See text for more details.

Results reported here prompted us to propose that TIPE proteins are the long-sought-after molecular pilot of leukocytes (Fig. 6). It has long been suspected that migrating cells may possess an internal compass that controls the directionality of migration^37–39^. Both PtdIns(3,4,5)P_3_ and small GTPases have been proposed to fulfil the role of the compass^37,38,40^. However, genetic mutation studies of PI3Ks indicate that directionality (but not speed) can still be maintained in the absence of PtdIns(3,4,5)P_3_, indicating that additional mechanisms may be involved^41^. Although small GTPases such as Rac control directionality, they are essential for supporting migration speed as well^40^. These observations have led to the proposition that the internal compass may not exist and cells may follow chemoattractant gradients by biased splitting of their protrusions^31^. Data reported here indicate that TIPE proteins specifically control the directionality of cell migration, with little effect on velocity.

Following chemokine receptor activation, TIPE proteins serve as the pilot of cell migration by steering cells along the chemokine gradient through at least two distinct mechanisms (Fig. 6). First, at the membrane-cytosol interface, PI3Ks alone may not be effective in catalyzing reactions of membrane-anchored phosphoinositides^42^. Because of its unique membrane-cytosol interfacial localization and its water solubility, TIPE-anchored PIP2 serves as a “primed’ or “presented” substrate for PI3Ks. Therefore, at the cell front, TIPEs promote leading edge formation by enhancing/exciting phosphoinositide signaling, leading to enhanced recruitment and activation of the downstream signaling molecules. Second, in the cytosol or away from the leading edge, the water soluble TIPEs serve as an inhibitor of Rac, preventing it from moving to the membrane as we reported^24^. This inhibitory mechanism is important for maintaining the polarized state of the cell, i.e., by preventing additional leading-edge formation. Thus, by regulating both PI3Ks and Rac, TIPE proteins confer the directionality of migration.

Although TIPE proteins are evolutionarily conserved in the Kingdom Animalia, with mammals having four closely related members, single cell organisms such as *Dictyostelium discoideum* have only one weakly related protein called GIP1 (G protein-interacting protein 1)^43^. Interestingly, GIP1 is also involved in regulating the directionality of migration of *Dictyostelium discoideum*^43^. *Dictyostelium discoideum* deficient in GIP1 has a severe defect in following the high concentration gradient of the chemoattractant cyclic adenosine monophosphate (cAMP). While mammalian TNFAIP8 and TIPE2 redundancy is required for maintaining T cell directionality, it remains to be determined whether other molecules play a redundant role with GIP1 in *Dictyostelium discoideum*.

In summary, we discovered that TIPE family of proteins controls lymphocyte migration and deployment in healthy and diseased animals. These results may not only advance our understanding of the biology of lymphocyte trafficking but also help develop new TIPE-based strategies to treat lymphocyte-related diseases. For example, agents that block TIPE function could be effective for treating inflammatory diseases such as MS. However, the same agents could also reduce the host’s ability to mobilize immune cells to fight against infectious microbes. Therefore, future studies are needed to evaluate the therapeutic and adverse effects of TIPE-blocking agents for the treatment of inflammatory diseases.

## Supplementary information

Supplementary Information.

Supplementary Information 2.

Supplementary Information 3.

Supplementary Information 4.

Supplementary Information 5.

## Data Availability

The data that support the findings of this study are available from the corresponding author upon request.
