# Supplementary material for: The TIPE Molecular Pilot That Directs Lymphocyte Migration in Health and Inflammation
Source: Sci Rep. 2020 Apr 20;10:6617. doi: 10.1038/s41598-020-63629-w (PMC7170861; doi:10.1038/s41598-020-63629-w)
Supplement: Supplementary file 1 — Supplementary Information. [file 41598_2020_63629_MOESM1_ESM.pdf]

Supplementary Information

The TIPE Molecular Pilot That Directs Lymphocyte Migration in Health and Inflammation

Honghong Sun, Mei Lin, Ali Zamani, Jason R. Goldsmith, Amanda E. Boggs, Mingyue Li, Chin-Nien Lee, Xu Chen, Xinyuan Li, Ting Li, Brigid L. Dorrity, Ning Li, Yunwei Lou, Songlin Shi[1], Wei Wang, Youhai H. Chen

1. Supplementary Figures and Legends

2. Supplementary Video Legends

1. Supplementary Figures and Legends

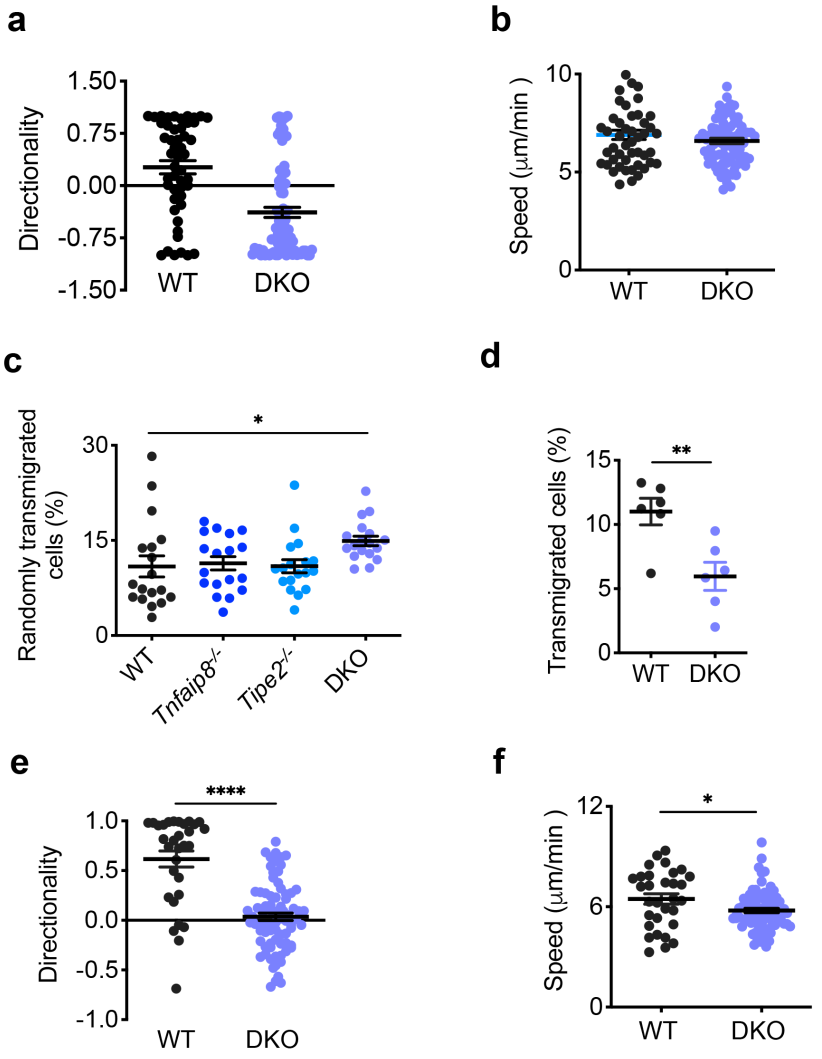

Supplementary Figure 1. Effects of TIPE deficiency on T cell and neutrophil migration. a, b, Directionality **(a)** and speed **(b)** of $CD4^+$ T cells from WT and DKO mice (5 mice per group), in the absence of added chemokines, as determined in the μ-slide migration assay. n=49 cells for WT, and 84 for DKO group. **c,** Percentages of transmigrated $CD4^+$ T cells of the indicated genotypes in the absence of added chemokines as determined in the transwell transmigration assay described in Fig. 1d (n=18 samples per group). **d,** Percentages of transmigrated $CD4^+$ T cells of WT and DKO groups (n=6 samples per group) in the presence of CXCL12. **e, f,** Directionality (**e**) and speed (**f**) of neutrophils from WT and DKO mice (4 mice per group), in response to CXCL1, as determined in the μ-slide migration assay. n=31 cells for WT, and 79 for DKO group. Values are mean ± s.e.m., and are pooled from two (**d**) or four (**c**), or are representative of three **(e, f)** or two (**a, b**) independent experiments. *P < 0.05; **P < 0.01; ****P < 0.0001 (Student's t-test (**a, b, c, d, e, f**))

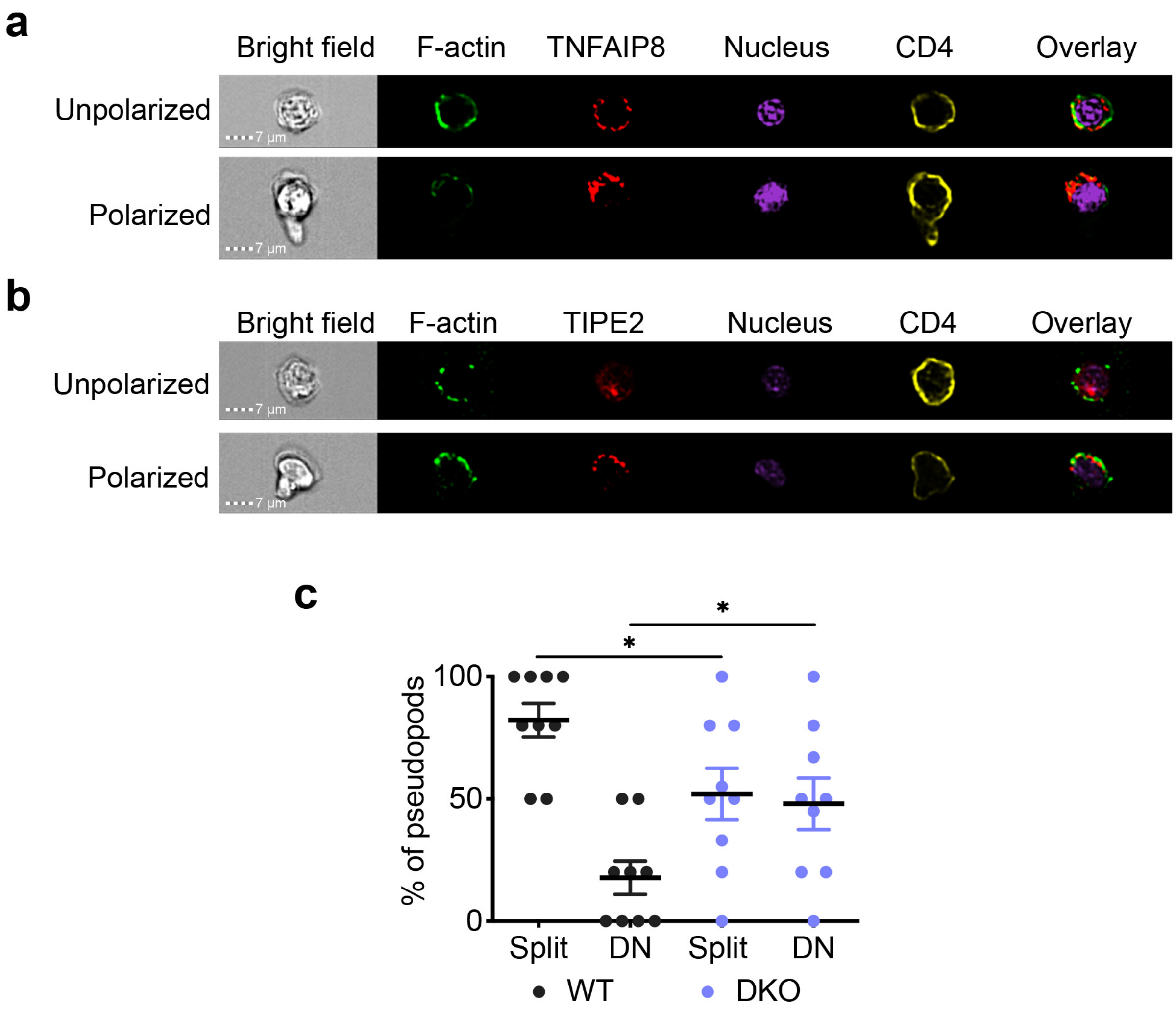

Supplementary Figure 2. TIPE protein polarization, and pseudopod formation in CD4$^+$ T cells. a, b, Visualization of TNFAIP8 **(a)** and TIPE2 **(b)** together with F-actin and CD4 in polarized and unpolarized WT CD4$^+$ T cells 10 min after CCL21 stimulation by confocal imaging flow cytometry. **c**, Percentages of pseudopods formed de novo (DN) or by splitting the existing pseudopods (with a splitting angle < 90°) of WT and DKO T cells 0 to 120 sec after point source stimulation with CCL21, calculated by live cell video microscopy; n=9 samples for each group. Values are mean ± s.e.m. (**c**). The experiments were repeated at least three times with similar results (**a-c**). *$P < 0.05$ (Student's *t*-test (**c**)).

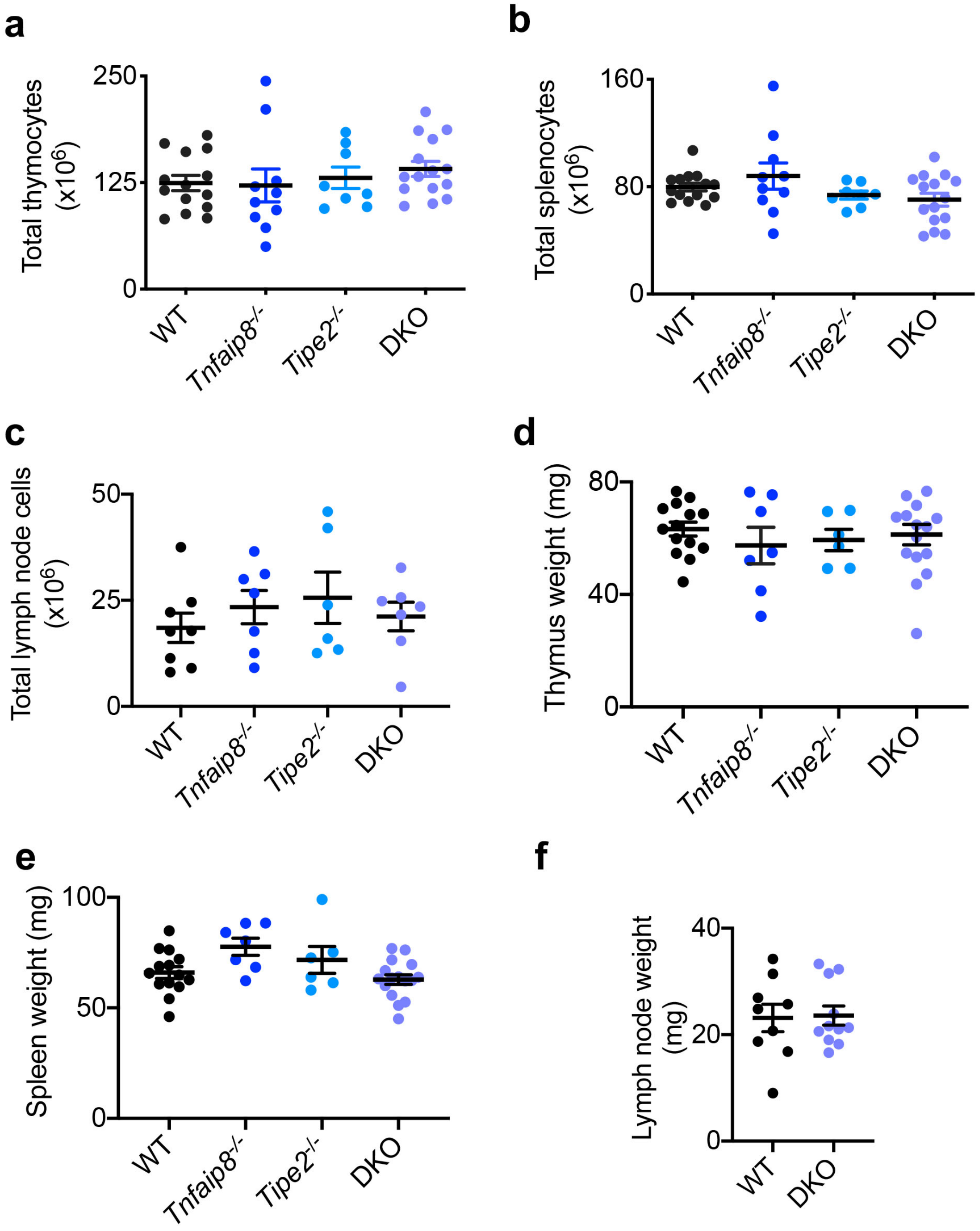

Supplementary Figure 3. Weights and total cell numbers of thymus, spleen and mesentery lymph node. a, b, Total numbers of thymocytes (**a**) and splenocytes (**b**) of each 8-week-old WT (n=14), *Tnfaip8*$^{-/-}$ (n=10), *Tipe2*$^{-/-}$ (n=8), and DKO (n=15) mouse. **c.** Total numbers of mesentery lymph node cells of WT (n=8), *Tnfaip8*$^{-/-}$ (n=7), *Tipe2*$^{-/-}$ (n=6), and DKO (n=8) mice. **d-f,** Weights of thymus (**d**), spleen (**e**), and mesentery lymph node (**f**) of the mice used in **a**-**c**. The values are mean ± s.e.m., and are pooled from three independent experiments (Student's *t*-test)(**a-f**).

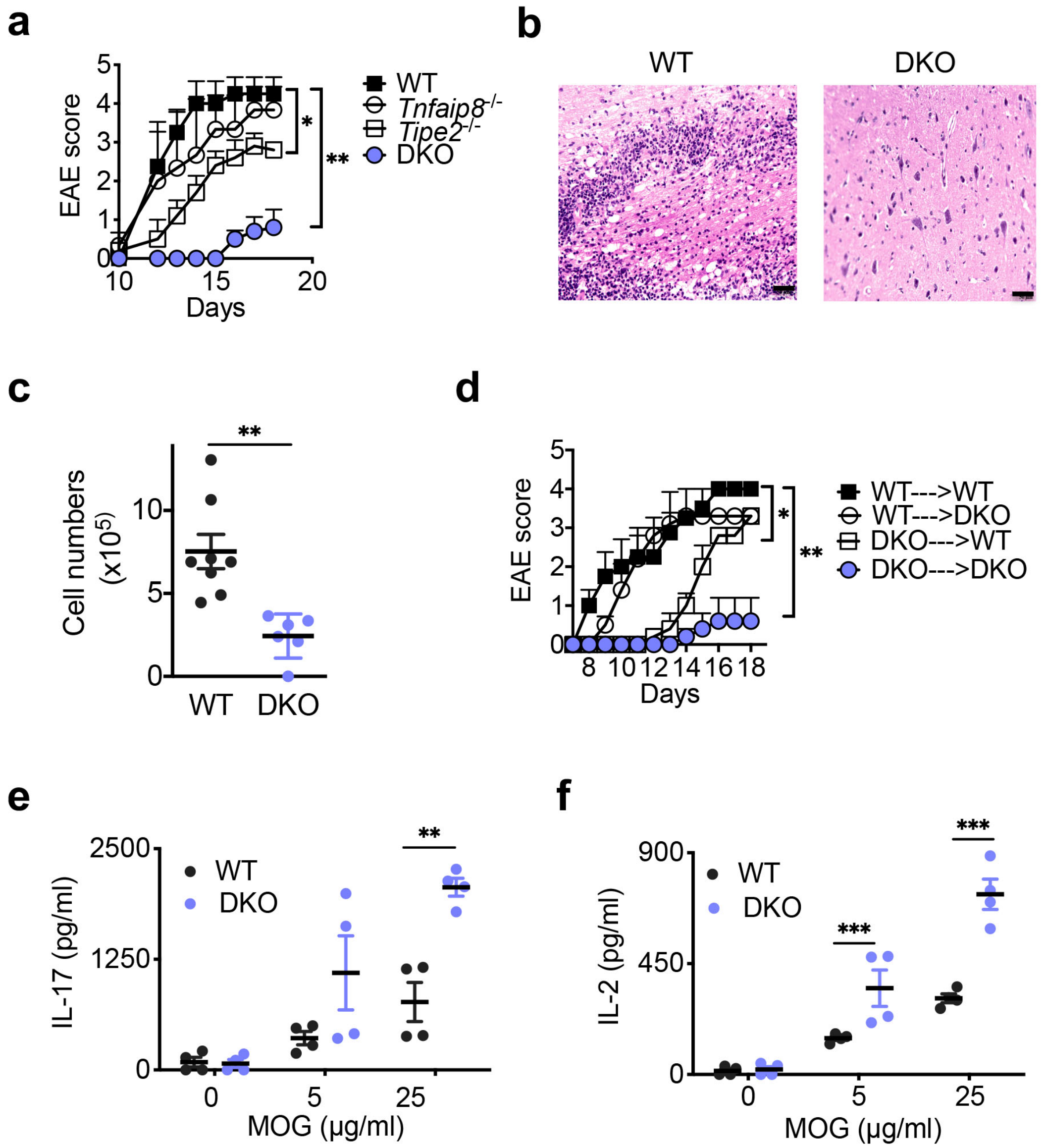

Supplementary Figure 4. The effects of TIPE deficiency on EAE, leukocyte infiltration of the nervous tissue, and T cell response to MOG. a, Clinical EAE scores of WT, *Tnfaip8*$^{-/-}$, *Tipe2*$^{-/-}$, and DKO mice (n=5 for each group) immunized with MOG35-55 peptide. **b,** Spinal cord sections stained with hematoxylin and eosin of WT and DKO mice. Scale bar = 50 μm. **c**, The numbers of leukocytes isolated from each spinal cord of WT (n=8) and DKO (n=6) mice 20 days after immunization with MOG. **d**, EAE scores of irradiated WT and DKO mice (n=5 for each group) that had received WT or DKO bone marrow cells before being immunized with MOG. **e, f,** The concentrations of IL-17A (**e**) and IL-2 (**f**) in the splenocyte cultures of WT and DKO EAE mice (n=4 per group), cultured with or without the MOG peptide for 24 h, as measured by ELISA. Data are representative of two (**d**) or three (**a, b**) independent experiments, or are pooled from two experiments (**c, e, f**). Values are mean ± s.e.m. **P* < 0.05; ***P* < 0.01; ****P* < 0.001 (Mann-Whitney *U* test (**a, d**) or Student's *t*-test (**c, e, f**)).

2. Supplementary Video Legends

Supplementary Video 1. Migration of WT T cells in response to CCL21. WT T cells were prepared and treated as described in Fig. 1a, and their migration in a μ-slide was recorded by time-lapse video microscopy. CCL21 was applied to the left side of the slide chamber.

Supplementary Video 2. Migration of DKO T cells in response to CCL21. DKO T cells were prepared and treated as described in Fig. 1a, and their migration in a μ-slide was recorded by time-lapse video microscopy. CCL21 was applied to the left side of the slide chamber.

Supplementary Video 3. Migration of WT neutrophils in response to CXCL1. WT neutrophils were prepared and treated as described in Extended Data Fig. 1c, and their migration in a μ-slide was recorded by time-lapse video microscopy. CXCL1 was applied to the top of the slide chamber.

Supplementary Video 4. Migration of DKO neutrophils in response to CXCL1. DKO neutrophils were prepared and treated as described in Extended Data Fig. 1c, and their migration in a μ-slide was recorded by time-lapse video microscopy. CXCL1 was applied to the top of the slide chamber.
